# Sequence characterization and polymorphism of melanocortin 1 receptor gene in some goat breeds with different coat color of Mongolia

**DOI:** 10.5713/ajas.18.0819

**Published:** 2019-02-07

**Authors:** Onolragchaa Ganbold, Prabuddha Manjula, Seung-Hwan Lee, Woon Kee Paek, Dongwon Seo, Munkhbaatar Munkhbayar, Jun Heon Lee

**Affiliations:** 1 Division of Animal and Dairy Science, Chungnam National University, Daejeon 34134, Korea; 2 Department of Biological Science, Mongolian National University of Education, Ulaanbaatar 210685, Mongolia; 3 Division of Research and Promotion, National Science Museum of Korea, Daejeon 34143, Korea

**Keywords:** Coat Color, Melanocortin 1 Receptor (*MC1R*), Missense Mutation, Mongolian Goat

## Abstract

**Objective:**

*Extension* and *Agouti* loci play a key role for proportions of eumelanin and pheomelanin in determining coat color in several species, including goat. Mongolian goats exhibit diverse types of coat color phenotypes. In this study, investigation of the melanocortin 1 receptor (*MC1R*) coding region in different coat colors in Mongolian goats was performed to ascertain the presence of the *extension* allele.

**Methods:**

A total of 105 goat samples representing three goat breeds were collected for this study from middle Mongolia. A 938 base pair (bp) long coding region of the *MC1R* gene was sequenced for three different breeds with different coat colors (Gobi Gurwan Saikhan: complete black, Zalaa Jinstiin Tsagaan: complete white, Mongolian native goat: admixture of different of coat colors). The genotypes of these goats were obtained from analyzing and comparing the sequencing results.

**Results:**

A total of seven haplotypes defined by five substitution were identified. The five single nucleotide polymorphisms included two synonymous mutations (*c.183C>T* and *c.489G>A*) and three missense (non-synonymous) mutations (*c.676A>G*, *c.748T>G*, and *c.770T>A*). Comparison of genotypes frequencies of two common missense mutions using chi-sqaure (*x*^2^) test revealed significant differences between coat color groups (p<0.001). A logistic regression analysis additionally suggested highly significant association between genotypes and variation of black versus white uniform combination. Alternatively, most investigated goats (60.4%) belonged to H2 (TGAGT) haplotype.

**Conclusion:**

According to the findings obtained in this study on the investigated coat colors, mutations in *MC1R* gene may have the crucial role for determining eumelanin and pheomelanin phenotypes. Due to the complication of coat color phenotype, more detailed investigation needed.

## INTRODUCTION

Changes in coat color are one of the first hallmarks of domestication [1], and human interest in coat colors make it more diverse [2]. On the other hand, approximately 350 loci that influence mammalian goat color were discovered previously (most of them first identified in lab mice and rats) [2]. In addtion, the coat color is an important trait in breed recognition for numerous domestic mammalian and avian species. Recently, a large number of coat color phenotypes have been described in different domestic animals [3]. For an example, in goats, several variations of coat color were observed such as, black, red, white, spotted, blue and pied. From an economical perspective, coat color traits play an important role in wool and cashmere production [4].

Among the genes that are known to be involved in pigmen tation, tyrosinase-related protein (*TYRP1*), Melanogenesis associated transcription factor (*MITF*), and KIT ligand (*KITLG*) genes could directly or indirectly affect the production of two types of pigments, pheomelanin (yellow/red or white color) and eumelanin (dark color) in mammals [5]. Furthermore, these two melanins are synthesized by melanocytes thus, color depends on the presence, distribution and biochemical activities of the melanocytes [3]. The *Extension* and *Agouti* are the main loci that primarily affect the proportions of eumelanin and pheomelanin. The extension locus encodes the melanocortin 1 receptor (*MC1R*) gene that has a seven transmembrane domains protein belonging to the G protein coupled receptors [6].

The non-synonymous mutations (missense and nonsense) of *MC1R* gene directly affect gain or loss of function that determine the dominant or partially dominent black/dark and recessive or partially recessive red/yellow or white coat color phenotypes, respectively. More evidences on these mutations were described in several mammals, including, mice [5], humans [7], sheep [8], cattle [9], pigs [10], horses [11], dogs [12], foxes [13], and rabbits [14].

Historically, Mongolians have reared relatively smaller pop ulations of goat that accounted for ~17% of total domestic animals in the country before and during Soviet Union rule. However, the goat breeds became the most economical animals in Mongolia after 1990s democratic revolution, because of a rapidly increased cashmere price (from ~$4.0 up to approximately $40.0 for a kilo) [15]. Consequently, following this phenomenon, the number of goats rapidly increased since 1990s, for example, 5.1 millon goats were counted in 1990, followed by 10.3 for 2000 and 19.2 million head of goats in 2013 [15]. In 2017, Mongolia had 27.3 million goats (41.3% of domestic animals) [15]. These economically important goats produced one of the major export products, cashmere, and Mongolia became the second largest cashmere producer in the world, second only to China [16]. Since the goat coat color is strongly linked to the cashmere products in Mongolia, the light colored cashmere that comes from goats with light phenotype (e.g. white or red) receive higher price. Mongolian goats show a remarkable variety of coat colors varying from white to black, together with several other colors, including red. Although, there is conspicuous diversity, there are no studies avialable that document coat color pigmentation of Mongolian goats, including economically important white coat color phenotype.

Therefore, better understanding the molecular background of Mongolian goats’ coat color (especially white, black, and red) is very important. The majority of previous studies [3,17, 18] focused on mutations in *MC1R* gene primarily controling black and white coat color variation. For this reason, we have separately done an association test for these two coat colors. However, some previous studies also suggested that the existence of the red (at least red-head or neck) phenotypes was highly associated wth a recessive *e* allele in some breeds [17,18]. Further study may contribute to improve the understanding of goat coat color genetics that is vital for designing a new goat breeding program in Mongolia, as well as improving the quality (with desired light color) of fiber product for exportation. Hence, in the present study, we identify and investigate polymorphisms (single nucleotide polymorphism [SNP]) in the exon of caprine *MC1R* gene that plays a central role in regulation of animals coat color formation, to examine presence and potential effect of this locus in different coat color groups in Mongolian native goat (MNG), Gobi Gurwan Saikhan (GGS), and Zalaa Jinstiin Tsagaan (ZJT) goats. We also hypothized that the predominant red coat color is produced by homozygous *e*/*e* alleles, and black coat color results from dominant allele *E^D^*, while wild type allele *E*^+^ might produced the miscellaneous coat colors in Mongolian goat populations.

## MATERIALS AND METHODS

### Animal sampling and DNA extraction

**Figure 1:**
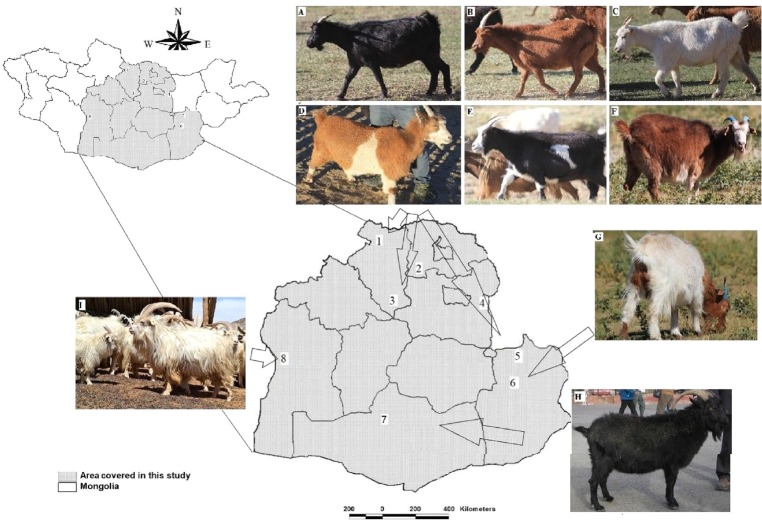
Photos of investigated goats with different coat colors and sampling localities in middle Mongolia. For coat color: Mongolian native goats (MNG: A, black; B, red; C, white; D, red pied; E, black pied; F, white faced red; G, red headed white), Gobi-Gurvan Saikhan (GGS: H, black), and Zalaa Jinstiin Tsagaan (ZJTs: I, white). For populations: 1 to 6 for MNG populations: 1. Bulgan (local MNG, n = 10), 2. Lun Og (local MNG, n = 10), 3. Lun Tsagaan-Uul (local MNG, n = 18), 4. Dalanjargalan (local MNG, n = 15), 5. Ikh Nart (local MNG, n = 22), 6. Nomgon (local MNG, n = 10), 7. Omnogobi-Bulgan (GGS, n = 10), and 8. Jinst (ZJTs, n = 10).

All sampling procedures in this study was adhered to the guidelines of Ethic Committee of Veterinary Research Institute of Mongolia (ref. N 256/2010). Goats samples were collected across the middle Mongolian region incorporating desert, dry steppe, mountain steppe and dense forest natural zones with decreasing temperature going northward (including partial northern, central and southern Mongolia) (Figure 1). A total of 105 goat samples from three breeds were used for this study; 85 MNG (accounted for >95% of total goats in Mongolia), 10 GGS and 10 ZJT. The GGS and ZJT breeds were introduced from Soviet-bloc countries [19] and have become well adapted in the desert region in southern Mongolia, mostly reared in Bulgan soum (or village) of Omnogobi province, and Jinst soum in Bayankhongor province, respectively. While MNG is the most widely distributed and common across Mongolia. We sampled from eight different populations; six populations for MNG and one unique population for GGS and ZJTs, respectively. The phenotypes (coat color) of these animals are shown in Figure 1. The GGS and ZJT goat breeds’ status were characterized by their uniform black and white coat colors [19]. Whereas, in MNG goats variable coat colors, such as, red and black color, white, blue, white spotted on the body or face or red-headed white, or various combinations of these colors have been observed (Supplementary Table S1).

Eight mL of blood samples were collected from MNG and GGS goats, whereas, tissue samples from ear were collected from ZJT goats. Blood (with vacuum tubes inclduing ethylenediaminetetraacetic acid) and ear (with 70% ethanol) samples were stored in car portable freezer (−4°C to −12°C) during prolonged field work. Blood samples of Mongolian goats were preserved on FTA classic cards (Whatman: Lot No.9767339, Maidstone, UK) in order to transfer samples to Korea. Genomic DNA was extracted from three punches of filter paper (FTA, 2 mm punches) using MagMax DNA Multi-Sample Ultra Kit (TermoFisher Scientific, Baltics UAB V.A. Vilnius, Lithuania) using an extraction protocol with a magnetic beads following the manufacturer’s instructions.

### Polymerase chain reaction amplification and sequencing of *MC1R* gene

A long exon (938-bp) of the *MC1R* gene of goat was amplified to identify the polymorphisms. A pair of primers (Forward: 5′-CTG AGA GCA AGC ACC CTT TC -3′; Reverse: 5′-GTC CTG TGA TTC CCC TCT CA -3′) for caprine *MC1R* polymerase chain reaction (PCR) amplification and sequencing was designed with Primer3Plus tools (online available at: https://primer3plus.com/cgi-bin/dev/primer3plus.cgi) used previously and published as goat reference sequence (Genbank accession number: Y13958) [20]. The PCR amplifications for goat *MC1R* gene was performed by using thermal cycler C1000 (Bio-Rad laboratories Inc., Hercules, CA, USA) system in a volume of 20 μL that contained 2.0 μL genomic DNA (15 ng/μL), 0.8 μL of each primer (10 pmoles/μL), 2.0 μL 10× reaction buffer, 1.6 μL dNTP, 0.2 μL prime Taq DNA polymerase, and 12.6 μL distilled water for adjusting final PCR master mix volume. The reaction was carried out with initial denaturation for 3 min at 94°C, followed by 35 cycles of denaturation for 30 s at 94°C, annealing for 30 s at 58°C, extension for 45 s at 72°C, and final extension at 72°C for 5 min. The amplified DNA fragments were detected on 1.5% agarose gel, and size checked using 100 bp DNA ladder (GeNetBio, Daejeon, Korea), and photographed under UV light (in Printgraph, Atto Corporation, Tokyo, Japan). For sequencing, PCR amplicons were purified with PCR purification kit (GeNetBio, Korea) following the manufacturer’s recommendation. All 105 samples were sequenced in this study (Cosmogenetech Company [www.cosmogenetech.com]).

### Single nucleotide polymorphism identification and genotyping

A total of 105 novel sequences of Mongolian goats (85 MNG, 10 GGS, and ZJTs, respectively) were initially assembled in BioEdit V7.2.5 [21] and aligned with the Clustal W multiple aligning tools [22] for identification of SNPs and further analysis. The SNPs were identified with the Clustel W aligner and DnaSP v.5.10.01 [23]. The variant effect predictor (VEP) in Ensemble web tool (http://asia.ensembl.org/Tools/VEP) was used to obtain the information of SNPs’ nomenclature and prediction of consequences of variants on the protein sequence (e.g. synonymous, missense and non-sense) based on NCBI accession number Y13958 as reference. The Mongolian goat *MC1R* genotypes were obtained from analyzing and comparing the obtained sample using a sequencing electropherogram approach (Supplementary Figure S1a).

### Statistical analysis

The DnaSP V5.10.01 (DNA sequence polymorphism, [23]) was applied for estimating the diversity parameters, including haplotype diversities (*H*d), and nucleotide diversities (Pi), as well as number of haplotypes (H), number of segregating sites (S), average number of pairwise different (K), and haplotype file were also generated for further haplotype network analysis. The molecular phylogenetic tree was constructed by maximum-likelihood (ML) method based on Tamura-Nei model [24] in molecular evolutionary genetics analyser V7 (MEGA) [25]. We drew the median-Joining (MJ) network using NETWORK V5.0.0.1 to reveal genetic relationships among *MC1R* haplotypes in Mongolian goats [26].

Alternatively, the online tool PROVEAN V1.1 (Protein Variation Effect Analyzer, avialable at: http://provean.jcvi.org/seq_submit.php) was used to predict amino acid substitution effect on protein function. If the Provean provided a value that was equal to or below a predefined threshold (e.g. −2.5), the protein variant was predicted to have a deleterious effect on its function, while the protein variant was predicted to have a neutral effect when the score was above the threshold [27]. Finally, the PROTTER v1.0 online tools (http://wlab.ethz.ch/protter/#) [28] was used to predict the secondary structure of the caprine *MC1R* protein using reference amino acid sequence (CAR82366) [3].

The protein sequence for *MC1R* gene (Genbank accession number: CAR82366) of *capra hircus* was acquired from National Center for Biotechnology Information (NCBI) and blast in Clustal W tools [22] against protein sequences, that also were obtained from NCBI: *ovis aries* (SBU12237), *bos taurus* (NP_776533), *equus caballus* (NP_001108006), *Sus scrofa* (ALN40197), *Homo sapiens* (NP_002377), *Felis catus* (NP_ 001009324), *Canus l. Familiaris* (NP_001014304), *Oryctolagus cuniculus* (CBJ17605), *Mus musculus* (NP_032585), *Gallus gallus* (AGY49247), *Anas platerhynchos* (NP_001297734), *Danio rerio* (NP_851301).

For the purpose of statistics, coat colors were divided into three main groups: i) Red (n = 50, complete red, white-faced red and red-pied), ii) Black (n = 31, complete black and black-pied), and iii) White (n = 18). These three color groups also are dominant in Mongolian goat populations. Six goats were excluded from association test due to low frequency of other minor coat color expression. In this study, the two main statistical tests were used to ascertain association between genotype and phenotype, a logistic regression for coat color groups of black versus white and a chi-square (*x*^2^) test for three groups of coat color, and these tests were implemented in R version 3.5.1.

## RESULTS AND DISCUSSION

### Sequence variation and haplotypes of *MC1R* gene in Mongolian goat populations

The PCR amplification produced 1,020-bp fragments of coding region (CDS). After initial sequence editing, the 938-bp long fragments were utilized in downstream analysis. The obtained sequences represented three different breeds from eight independent populations.

Analysis of the *MC1R* gene sequences revealed a total of five substitutions in Mongolian goat populations. The number of polymorphic sites for MNG, GGS, and ZJTs goats were comparable. In addition, the overall estimated nucleotide (Pi) diversity for investigated goat populations was 9.7×10^−4^ (Supplementary Table S2). Alternatively, Pi value was estimated for the three coat color groups. According to diversity estimation, the highest Pi was observed for white animals (11.6×10^−3^) (Supplementary Table S3). Observed diversity indices may suggest that black and white uniformed animals had higher polymorphism than predominant red.

**Figure 2:**
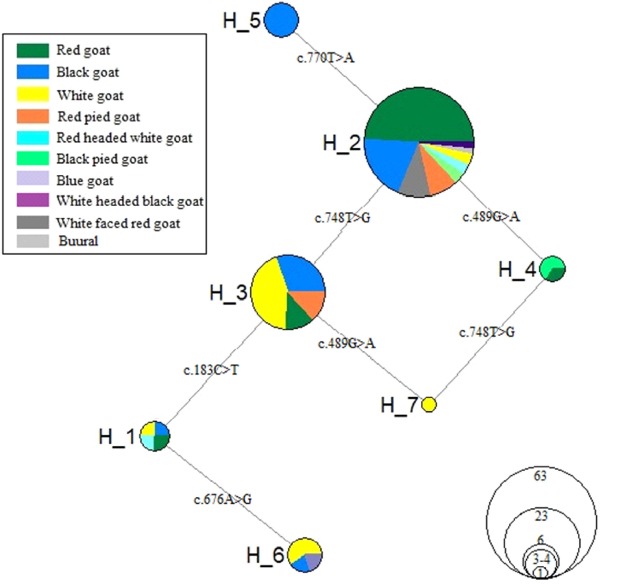
Median-joining (MJ) network illustrating relationships among the seven haplotypes in Mongolian goats. Each polymorphic sites (single nucleotide polymorphisms) are shown. The majority of the investigated goats were belonged to the H2 that dominanted by the red uniformed goats. NETWORK V5.0.0.1 was used to construct MJ.

A total of seven *MC1R* gene haplotypes were defined by five polymorphic sites in Mongolian goats. The similar number of *MC1R* haplotypes and much higher number of agouti signaling protein haplotypes were found in different goat populations [3,29,30]. The MJ network and ML tree shows their relationships (Figures 2, Supplementary Figure S2). According to these clusters, H2 (TGAGT), H3 (TGATT), and H5 (TGAGA) were closely clustered compared to others (Supplementary Figure S2), while H6 (CGGTT) was separated by three mutation steps (*c.748T>G*, *c.183C>T*, and *c.676A>G*) from central H2 (Figure 2). In addition, Table 1 reports haplotype frequencies and their distributions among Mongolian goat populations. The H2 showed the highest frequency (60.4%) in two breeds of goat (or populations; without ZJTs) including most of the recorded phenotypes (9 coat colors), followed by H3 (21.6%), while relatively low number of animals (from 1 to 6) were associated to the remainder of haplotypes (Table 1, Figure 2). In particularly, H7 (TAATT) acounted for only 1 white animal. Intrestingly, one haplotype (H5) was unique for black goats, whereas the majority of red goats (86.1% of total red group) belonged to the main *MC1R* haplotypes (H2), white predominant in H3 (55.5%) and H6 (22.2%), and black goats (50.0%) goat predominant in H4 (TAAGT) (Figure 2). According to the findings of this study, unique H5 (TGAGA) may have some relationship with black uniform, unfortunately there were a lack of such animals sample.

### Identification of mutations

**Figure 3:**
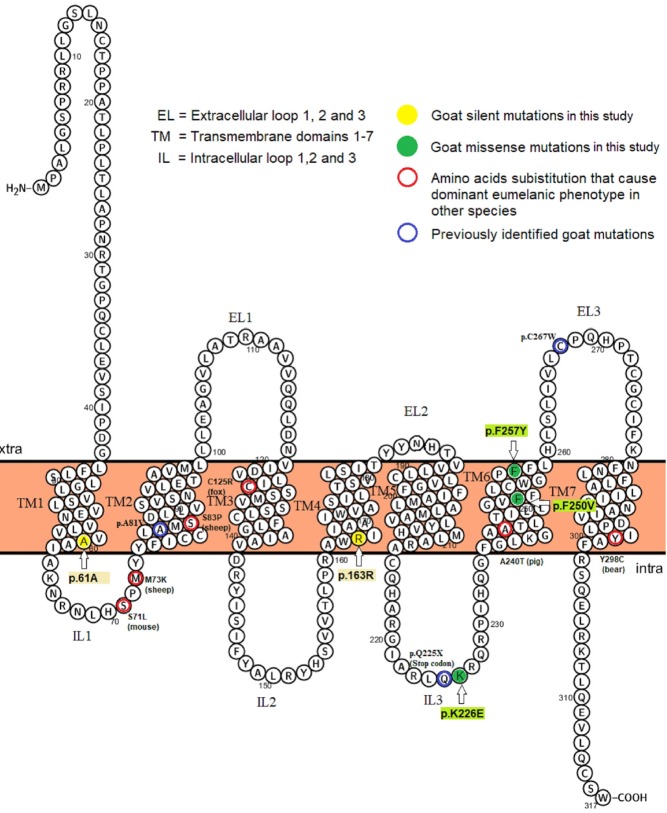
2D structure of the goat melanocortin 1 receptor (*MC1R*) amino acid sequence illustrating identified substitution in goat (this and previous studies) and other mammals [35]. Blue circle signs represent mutations in Fontanesi et al [3]. Four of all five single nucleotide polymorphisms were located in the transmembrane domains (TMs) while one in the intracellular loops-3 (IL3). 2D structure predicted in the PROTTER v1.0 online tools (http://wlab.ethz.ch/protter/#)[25].

The CDS region in goat *MC1R* gene encodes a protein of 317 amino acids that was similar to *bos taurus* and *ovis aries* with 97% and 99% identity, respectively (Figures 3, Supplementary Figure S1b). A total of five SNPs (polymorphic sites) were identified upon aligning the obtained sequences (CDS) of Mongolian goats (Table 2). Two out of five SNPs were identified as synonymous mutations (*c.183C>T*, *c.489G>A*), while the remaining three were missense mutations (non-synonymous), *c.676A>G*, *c.748T>G*, *c.770T>A* causing the *p.K226E*, *p.F250V*, and *p.F257Y* amino acid substitutions in transmembrane domains, TM1, TM4, TM6, and intracellular loops-3 of the *MC1R* protein (Table 2, Figure 3). Meanwhile, lysine (at 226) and phenylalanine (at 250 and 257) were highly conserved in the aligned melocortin 1 receptor in the amino acid sequences of diverse mammalian and avian species (Supplementary Figure S1b).

Among the identified five SNPs, three SNPs were previ ously described, namely, synonymous mutation *c.183C>T* and missense mutation *c.748T>G* by Fontanesi et al [3], and *c.676A>G* by Wu et al [17] and Javanmard et al [31]. Therefore, SNPs *c.489G>A* and *c.770T>A* are uniquely observed in this study. The Provean score predicted the amino acids substitution effect on protein function (Table 2). The Provean score revealed that two of three missense mutations (*p.F250V* and *p.F257Y*) may have deleterious effects with −6.435 and −2.674 scores, respectively. Whereas, *p.K226E* mutation had a neutral effect on protein function with −1.566 score (Table 2). Fontanesi et al [3] reported that putative functional effect of missense *p.F250V* mutation on the protein was deleterious with a subPSEC score −5.998 (substitution position-specific evolutionary conservation). Moreover, the *p.F250V* seems to be a more deleterious effect, because of this mutation scored a higher functional effect score in this and a previous study [3]. The subPSEC score works in parallel with Provean score to predict functional effect on protein indunction. No functional effect score was avialable for previously described missense *p.K226E* mutation [17].

### Analysis of the single nucleotide polymorphisms in Mongolian goats with different coat colors

A total of 105 animals were genotyped to analyze polymorpism in caprine *MC1R* gene. In addition, all of these animals’ phenotypes (coat color) were recorded during field sampling. Supplementary Table S4 reveals mutated allele and genotype frequencies for five identified polymorphic sites on the three phenotypic groups in Mongolian goats. Here, we further highlighted two common missense mutations (*c.676A>G* and *c.748T>G*), none for their two synonymous, and a very rare missense mutaion (*c.770T>A*). We performed a chi-sqaure test to assess genotype frequency differences among the three coat color groups, while a logistic regression for association of black versus white phynotypes and genotypes additionally.

For misse nse *c.676A>G* (*p.K226E*) mutation, frequencies of genotypes AG and GG was significantly greater in white (*x*^2^ = 39.1; d.f = 4; p<0.0001), while the AA genotypes are present in both red and black (Supplementary Table S4). In addition, dominant allele A (*E^D^*), frequency also was significantly higher, especially in the red group (see, Supplementary Table S5; Figure S3). In a previous study, Wu et al [17] found a higher frequency of GG genotype for the majority of investigated animals (dominated by complete white), while only *Boer* (red-headed white) goats carried AA genotype for *c.676A>G* mutation. Further, the authors highlighted that G allele in this mutation may be a loss of function mutation associated with complete white goat coat colors [17]. Whereas, Javanmard et al [31] found the higher frequency of AA genotype in eumelanin phenotypes.

Whereas, black and red, particularly red group accounted for higher frequencies of mutated allele (G) (84%) than white had for *c.748T>G* (*p.F250V*) mutation. However, genotype frequencies varied significantly among the groups, and homozygous genotype GG significantly higher in the red and black (*x*^2^ = 34.3; d.f = 4; p<0.0001) (Supplementary Table S4). It seems mutated type allele (G, or recessive e) may contribute to the red phenotypes in Mongolian goats (Supplementary Tables S4, S5). In contrast, Fontanesi et al [3] reported that goats with black eyed-white and black or brown coats were found to have a higher frequency of GG genotype (0.94% and 1.0%, respectively), while red goats (Derivata di Siria) had only 0.26% mutated allele for mutation *c.748T>G*.

Additionally, the main two color variations in mammals that are controlled by mutations in *MC1R* gene are black and white. A logistic regression also revealed a highly significant values for association between genotypes and these two phenotypes (p<0.05). Detailed information regarding the results between white and black individuals using logistic regression analysis were illustrated in Table 3. Therefore, based on the findings obtained from this study (association tests), the *Extension* locus in goat might play in some active role in balck versus white coat color varation, at least in the three investigated breeds in this study (white animals from MNG and ZJT; black from MNG and GGS).

Unfortunately, the most rare missense mutation *c.770T>A* (*p.F257Y*), which may contribute to the black coat color in Mongolian goats, effects could not be positively determined due to only six complete black goats (animals from both MNG and GGS; mutated allele frequency 0.11%) were heterozygous in this mutation (Supplementary Table S5). Moreover, five of these six goats belong to H5 haplotype (TGAGA). To reach a definitive conclusion, more animals need to be sampled.

Previously, some mutations at the extension locus that af fected different coat colors in different mammalian species such as, in goat [3], sheep [8,31], cattle [9], pigs [10], horses [11], dogs [12], rabbits [14] were reported. Whereas, in goat extension locus, Fontanesi et al [3] reported higly significant associations (Fisher exact test, p<0.001) between amino acid substitution and black coat color and red spotted white goats for *c.801C>G* missense and *c.673T* nonsense mutations, respectively. Unfortunately, the aforementioned mutations were not recorded in this study. In particularly, Fontanesi et al [3] emphasized the nonsense mutation (*c.673T*)’s alteration that resulted for shorter *MC1R* protein (Figure 3).

Interestingly, in this study two common missense muta tions (*c.676A>G* and *c.748T>G*) were expressed in animals with white uniform (did not influence any melanin) tended to carry a higher frequency of heterozygote genotypes (0.72% and 0.66%, respectively), this suggested that these mutations may have effect white coat colors. However, the determination of white coat color followed the classical rule of epistatic effects with Agouti locus, especially wild type allele (A^wt^) for this locus (or masked other unknown loci) [3,31].

As our previous hypothesis, the predominant black and red coat colors may carry *E^D^* and *e* alleles at extension locus, respectively. However, the present study did not completely support the hypothesis, due to conserved putative alleles not being observed in all loci. All three missense mutations gave different frequency patterns for normal and mutated alleles, and this pattern resulted in no expression of animals carrying wildtype allele (*E*^+^) in investigated Mongolian goats (Supplementary Table S5). This was also likely due to animals carrying such allele were not included in this study. In some previous studies, the complete wild type phenotypes were described in Camosciate delle Alpi and Saanen for *extension* and *agouti* (A^wt^) loci, respectively [3,32]. These wild type animals tended to carry a high frequency of normal alleles at the all loci. Fontanesi et al [3] investigated Derivata di Siria (red) goats in a previous study and the pheomelanin phenotype red, which they found and reported for clarification the dominance and incomplete penetrance of some “red alleles” at the extension locus.

Our findings as well as previous studies reported the highly conserved SNPs in wide range of goat populations may have an incomplete function on coat color phenotypes. This may be due to polygenic inheritance on coat color expression and masked effect of other loci. Therefore, previous studies [3,17, 31–34] emphasized that especially epistatic interaction of *Extension* and *Agouti* loci plays a significant role for this phenomenon. For an example, expression of *Agouti* alleles likely depends on at least one wild type copy (*E*^+^) putative allele at the *Extenstion* locus. More in detial, combination of dominant *agouti* (*A^wd^*) and recessive *extension* (*e*) has been proposed for black phenotype [35].

In conclussion, we report SNPs in the *MC1R* gene of Mongolian goats in this study, and looked for their association with genotypes. Some of our findings on coat colors suggested that mutations in *MC1R* gene may have some role for determining coat color phenotypes. However, according to entire results obtained in this study also demonstrated that this locus is probably not the only factor for coat color trait. Further, the necessity of elucidate the genetic factors, which regulate the pigmentation of coat colors, especially interaction between the loci that associated with these traits were highlighted. Furthermore, the limitation for this study was a lack of sample from all registered breeds or strains of Mongolian goat, and this lack of samples may have resulted in the non-expression of previously reported and novel altered point mutations. Although, we believe that the largest number of the recorded coat colors of Mongolian goats were investigated in this study.

## Supplementary Data



## Figures and Tables

**Table 1:** Constructed haplotypes of goat *MC1R* gene and their frequencies among investigated coat colors

Group	H1 CGATT n/%	H2 TGAGT n/%	H3 TGATT n/%	H4 TAAGT n/%	H5 TGAGA n/%	H6 CGGTT n/%	H7 TAATT n/%
Red	1 (0.25)	31 (0.50)	3 (0.13)	1 (0.33)	-	-	-
Black1)	1 (0.25)	13 (0.20)	7 (0.30)	-	5 (1.0)	1 (0.17)	-
White2)	1 (0.25)	2 (0.03)	10 (0.43)	-	-	4 (0.67)	1 (1.0)
RP	-	5 (0.07)	3 (0.13)	-	-	-	-
BP	-	2 (0.03)	-	2 (0.66)	-	-	-
WFR	-	6 (0.09)	-	-	-	-	-
RHW	1 (0.25)	2 (0.03)	-	-	-	-	-
Other	-	2 (0.03)	-	-	-	1 (0.17)	-
Total	4	63	23	3	5	6	1

*MC1R*, melanocortin 1 receptor; n, number of animals; %, frequency; RP, red pied; BP, black pied; WFR, red faced red; RHW, red headed white.

1) Indicating animals from MNG (Mongolian native goat) (n = 17) and GGS (Gobi Gurwan Saikhan) (n = 10).

2) Indicating animals from MNG (Mongolian native goat) (n = 8) and ZJTs (Zalaa Jinstiin Tsagaan) (n = 10).

**Table 2:** Identified polymorphism in *MC1R* gene of Mongolian goats

SNP	Location	Consequence	Codon	Amino acid substitution	PROVEAN score	Predicted effect
*c.183C>T*	18: 16105168	synonymous	GCC/GCT	p.61A	-	-
*c.489G>A*	18: 16105174	synonymous	AGG/AGA	p.163R	-	-
*c.676A>G*	18: 16105474	missense	AAG/GAG	p.K226E	−1.566	Neutral1)
*c.748T>G*	18: 16105733	missense	TTC/GTC	p.F250V	−6.435	Deleterious2)
*c.770T>A*	18: 16105755	missense	TTC/TAC	p.F257Y	−2.674	Deleterious

*MC1R*, melanocortin 1 receptor; SNP, single nucleotide polymorphism.

Predicted effects:

1) Neutral effects on protein function of point mutation in DNA sequence that are neither beneficial nor detrimental to the specific traits of an organism, while

2) deleterious effect that reduce protein function is detrimental or alteration for traits of an organism.

**Table 3:** Association analysis of two common missense mutations in goat *MC1R* gene between black and white coat colors of Mongolian goat populations

SNPs	Genotype			Estimate	Std. error	Z-value	Pr (> |z|)
*c.676A>G*	**AA**	**AG**	**GG**				
Black	23	7	1	−2.3949	0.7006	−3.419	0.00063
White	2	13	3				
*c.748T>G*	**TT**	**TG**	**GG**				
Black	2	7	22	2.1763	0.6655	3.270	0.00107
White	4	12	2				

*MC1R*, melanocortin 1 receptor; SNPs, single nucleotide polymorphisms, degree of freedom = 1.

## References

[1] Wilkins AS, Wrangham RW, Fitch WT (2014). The “Domestication Syndrome” in mammals: a unified explanation based on neural crest cell behavior and genetics. Genetics.

[2] Cieslak M, Reissmann M, Hofreiter M, Ludwig A (2011). Colours of domestication. Biol Rev Camb Philos Soc.

[3] Fontanesi L, Beretti F, Riggio V (2009). Missense and nonsense mutations in melanocortin 1 receptor (*MC1R*) gene of different goat breeds: association with red and black coat color phenotypes but with unexpected evidences. BMC Genet.

[4] Fontanesi L, Beretti F, Riggio V (2010). Sequence characterization of the melanocortin 1 receptor (MC1R) gene in sheep with different coat colors and identification of the putative e allele at the ovine *Extension* locus. Small Rumin Res.

[5] Chandramohan B, Renieri C, La Manna V, La Terza A (2013). The alpaca *agouti* gene: genomic locus, transcripts and causative mutations of eumelanic and pheomelanic coat color. Gene.

[6] Robbins LS, Nadeau JH, Johnson KR (1993). Pigmentation phenotypes of variant extension locus alleles result from point mutations that alter MSH receptor function. Cell.

[7] Valverde P, Healy E, Jackson I, Rees JL, Thody AJ (1995). Variants of the melanocyte–stimulating hormone receptor gene are associated with red hair and fair skin in humans. Nat Genet.

[8] Våge DI, Klungland H, Lu D, Cone RD (1999). Molecular and pharmacological characterization of dominant black coat color in sheep. Mamm Genome.

[9] Klungland H, Vage DI, Gomez-Raya L, Adalsteinsson S, Lien S (1995). The role of melanocyte-stimulating hormone (MSH) receptor in bovine coat color determination. Mamm Genome.

[10] Kijas JM, Wales R, Törnsten A, Chardon P, Moller M, Andersson L (1998). Melanocortin receptor 1 (MC1R) mutations and coat color in pigs. Genetics.

[11] Marklund L, Moller MJ, Sandberg K, Andersson L (1996). A missense mutation in the gene for melanocyte-stimulating hormone receptor (MCIR) is associated with the chestnut coat color in horses. Mamm Genome.

[12] Newton JM, Wilkie AL, He L (2000). Melanocortin 1 receptor variation in the domestic dog. Mamm Genome.

[13] Våge DI, Lu D, Klungland H, Lien S, Adalsteinsson S, Cone RD (1997). A non-epistatic interaction of agouti and extension in the fox, *Vulpes vulpes*. Nat Genet.

[14] Fontanesi L, Tazzoli M, Beretti F, Russo V (2006). Mutations in the melanocortin 1 receptor (*MC1R*) gene are associated with coat colors in the domestic rabbit (*Oryctolagus cuniculus*). Anim Genet.

[15] NSO (2017). National Statistics Office of Mongolia.

[16] Ganbold O, Lee SH, Dongwon S (2018). A review of population genetics research on domestic animals in Mongolia and recommendations for the improvements. J Anim Breed Genom.

[17] Wu ZL, Li XL, Liu YQ (2006). The red head and neck of Boer goats may be controlled by the recessive allele of the MC1R gene. Anim Res.

[18] Yang GL, Fu DL, Lang X (2013). Mutations in MC1R gene determine black coat color phenotype in Chinese sheep. Sci World J.

[19] Sandanjamts D, Minjigdorj B (2016). Registered livestock breeds in Mongolia.

[20] Klungland H, Røed KH, Neø CL, Jakobsen KS, Vage DI (1999). The melanocyte–stimulating hormone receptor (*Mci-R*) gene as a tool in evolutionary studies of artiodactyles. Hereditas.

[21] Hall TA (1999). BioEdit: a user-friendly biological sequence alignment editor and analysis program for Windows 95/98/NT.

[22] Thompson JD, Higgins DG, Gibson TJ (1994). CLUSTAL W: improving the sensitivity of progressive multiple sequence alignment through sequence weighting, position-specific gap penalties and weight matrix choice. Nucleic Acids Res.

[23] Librado P, Rozas J (2009). DnaSP v5: Software for comprehensive analysis of DNA polymorphism data. Bioinformatics.

[24] Tamura K, Nei M (1993). Estimation of the number of nucleotide substitutions in the control region of mitochondrial DNA in humans and chimpanzees. Mol Biol Evol.

[25] Kumar S, Stecher G, Tamura K (2016). MEGA7: Molecular evolutionary genetics analysis version 7.0 for bigger datasets. Mol Biol Evol.

[26] Bandelt HJ, Forster P, Rohl A (1999). Median-joining networks for inferring intraspecific phylogenies. Mol Biol Evol.

[27] Choi Y, Sims GE, Murphy S, Miller JR, Chan AP (2012). Predicting the functional effect of amino acid substitutions and indels. PLoS ONE.

[28] Omasits U, Ahrens CH, Müller S, Wollscheid B (2014). Protter: interactive protein feature visualization and integration with experimental proteomic data. Bioinformatics.

[29] Robbins LS, Nadeau JH, Johnson KR (1993). Pigmentation phenotypes of variant extension locus alleles result from point mutations that alter MSH receptor function. Cell.

[30] Adefenwa MA, Peters SO, Agaviezor BO (2013). Identification of single nucleotide polymorphisms in the agouti signaling protein (ASIP) gene in some goat breeds in tropical and temperate climates. Mol Biol Rep.

[31] Javanmard A, Arafnajad B, Arpanahi RA, Moradi MH (2015). Polymorphisms in melanocortin receptor 1 gene in goat breeds: a window for coat color controling mechanism. Iranian J Appl Anim Sci.

[32] Fontanesi L, Beretti F, Riggio V (2009). Copy number variation and missense mutations of the agouti signaling protein (*ASIP*) gene in goat breeds with different coat colors. Cytogenet Genome Res.

[33] Suhendra P, Rini W, Wayan TA (2016). Copy number variation of agouti signaling protein (ASIP) fragment and its relationship with coat color in indonesian goat breeds. Asian J Anim Vet Adv.

[34] Adalsteinsson S, Sponenberg DP, Alexieva S, Russel AJF (1994). Inheritance of goat coat colors. J Hered.

[35] Hepp D, Gonçalves GL, Moreira GRP (2012). Identification of the e allele at the Extension locus (MC1R) in Brazilian Creole sheep and its role in wool color variation. Genet Mol Res.

