# Supplementary material for: Sequence characterization and polymorphism of melanocortin 1 receptor gene in some goat breeds with different coat color of Mongolia
Source: Asian-Australas J Anim Sci. 2019 Feb 7;32(7):939–48. doi: 10.5713/ajas.18.0819 (PMC6601070; doi:10.5713/ajas.18.0819)
Supplement: Supplementary file 1 [file ajas-18-0819-suppl.pdf]

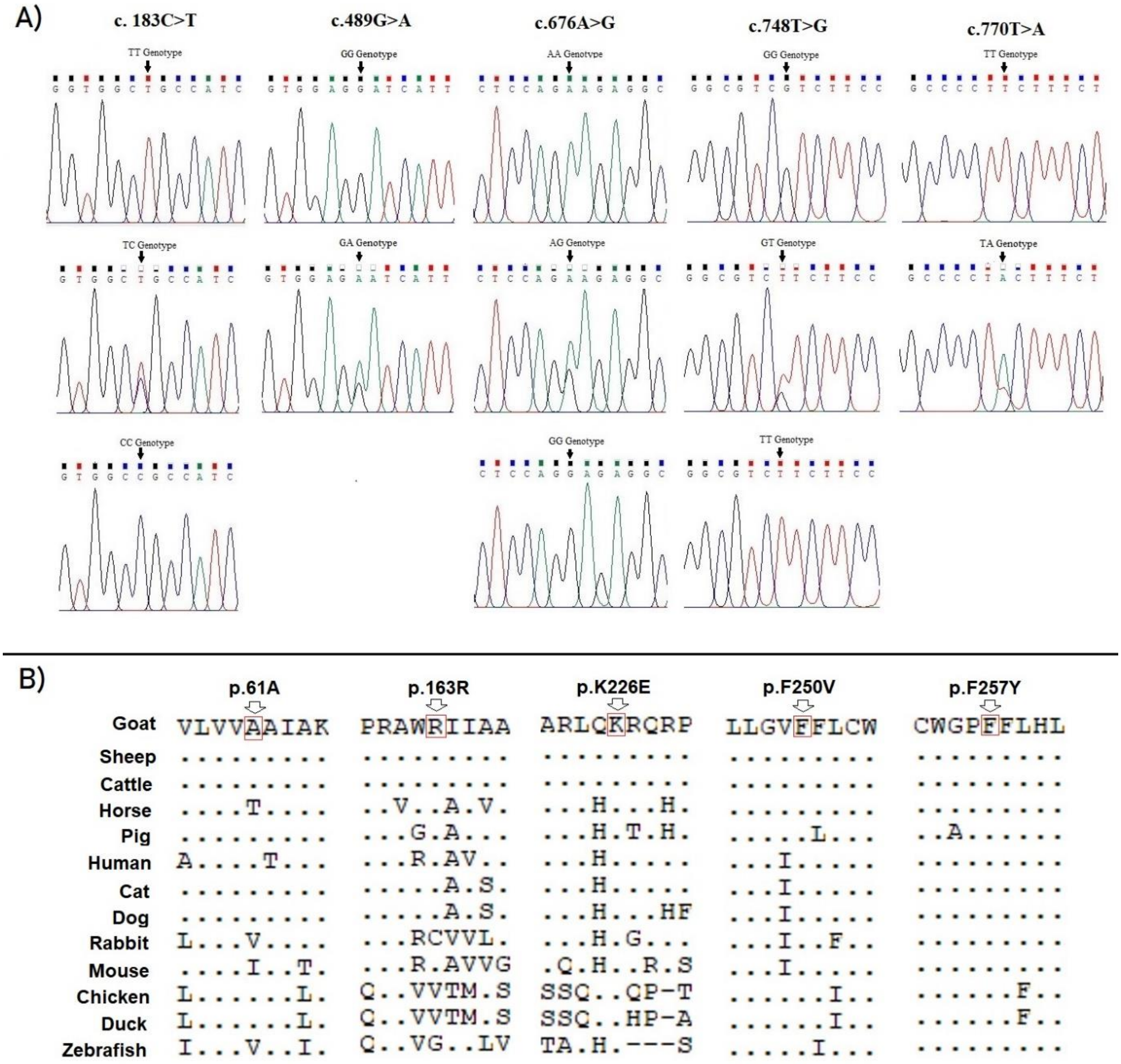

Figure S1. (A) Sequence electropherogams that showing both for the homozygous and heterozygous genotypes of identified five SNPs, (B) alignment of the MC1R protein regions around polymorphic sites with same region of other vertebrate species

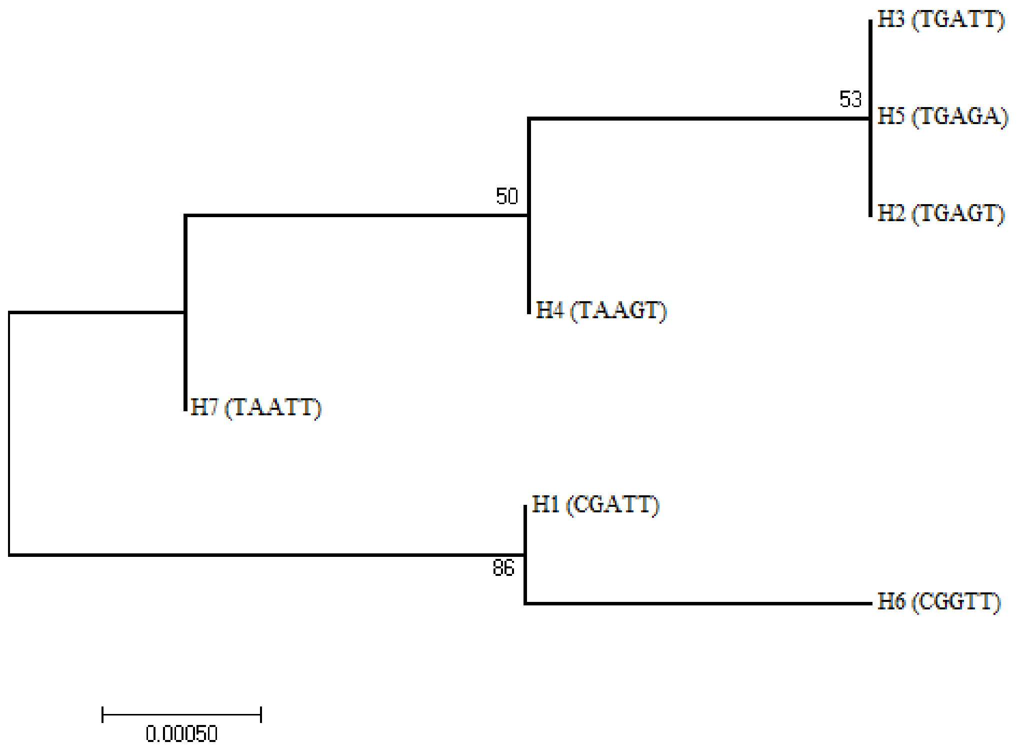

Figure S2. Maximum-likelihood (ML) tree has illustrated relationships of MC1R haplotypes in MNG

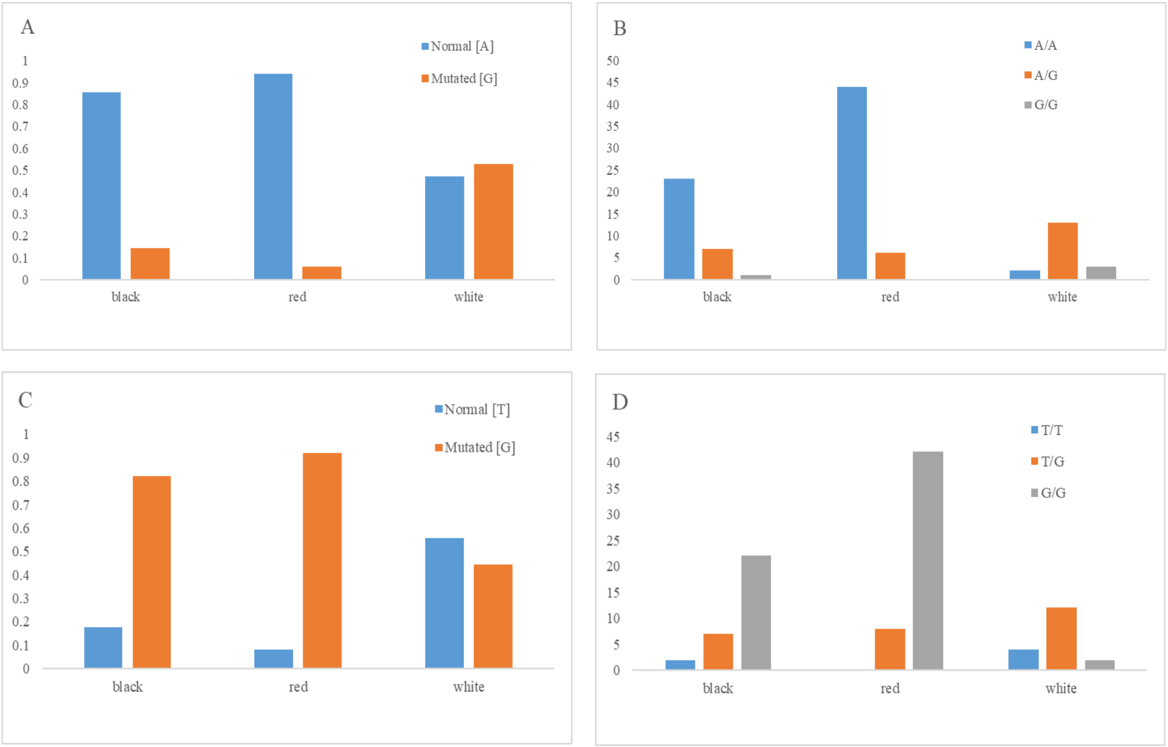

Figure S3. Allele and genotype frequencies of mutaion c.676A>G (A-B), and c.748T>G (C-D). Plot A and C showing allele frequencies, whereas B and D genotype frequencies.

Table S1. List of investigated coat color phenotypes in Mongolian native goats

Coat colour group	Breed	No. of animal	Status[2]
Red	MNG	37	Common
Black	MNG, GGS	27	Common
White	MNG, ZJTs	18	Moderate
Red pied	MNG	8	Moderate
Black pied	MNG	4	Rare
White-faced red	MNG	6	Moderate
Red-headed white	MNG	3	Rare
Other[1]	MNG	3	Very rare

MNG; Mongolian Native Goat, GGS; Gobi-Gurwan Saikhan, ZJTs; Zalaa-jinstiin tsagaan

[1]There are 3 different coat colors

[2]Status of occurrences of these phenotypes in Mongolian goat populations.

Table S2. MC1R gene polymorphisms and diversity of Mongolian goat populations

**Population/Breed**	**ID**	**n**[a)]	**H**[b)]	**Pi**[c)]	**S**[d)]	**K**[e)]
Mongolian native goat	MNG	85	6	6.3×10^{-4}	5	0.595
Gobi-Gurvan Saikhan	GGS	10	5	1.37×10^{-3}	4	1.289
Zalaa-Jinstiin Tsagaam	ZJTs	10	5	1.37×10^{-3}	3	1.278
Overall	-	105	7	8.9×10^{-4}	5	0.869

[a)] Number of investigated animals

[b)] Number of haplotypes

[c)] Nucleotide diversity

[d)] Number of polymorphic sites

[e)] Average number of pairwise differences

Table S3. MC1R gene diversity of main three coat color group in MNG

Group	No. of animal	H	Pi	S	K
Red[a)]	50	5	4.2×10^{-4}	3	0.364
White	18	5	11.6×10^{-3}	4	1.083
Black[b)]	27	5	10.7×10^{-3}	5	0.959

H= number of haplotypes, Pi= nucleotide diversity, S= number of polymorphic sites, K= average number of pairwise differences,

[a)] Group consisted of complete red, white-faced red and red pied animals

[b)] Group consisted of complete black and black pied animals

Figure S4. A significant chi-square test values for different distribution of genotypes of two common missense mutations in goat MC1R gene between coat color groups of Mongolian goat populations

**Groups**	**c.676A>G**			**p-value**	x^2	**c.748T>G**			**p-value**	x^2
	**AA**	**AG**	**GG**			**TT**	**TG**	**GG**		
Red	44	6	0			0	8	42		
Black	23	7	1	< *0.0001*	39.041	2	7	22	< *0.0001*	34.291
White	2	13	3			4	12	2		

MC1R, melanocortin 1 receptor, x^2 = chi-square test score, Degrees of freedom = 4

Table S5. Genotype distribution of identified SNPs in different coat color group of goats. Coat color groups that had more than 6 animals were reported.

Groups	No. of animal	SNP	Mutated allele frequency	Genotypes		
				**CC**	**TC**	**TT**
Red	50	**c.183C>T**	0.920	0.000	0.160	0.840
Black	31		0.822	0.064	0.225	0.709
White	18		0.444	0.222	0.667	0.111
				**GG**	**GA**	**AA**
Red	50	**c.489G>A**	0.010	0.980	0.020	0.000
Black	31		0.032	0.935	0.064	0.000
White	18		0.027	0.944	0.056	0.000
				**AA**	**AG**	**GG**
Red	50	**c.676A>G**[a), b)]	0.060	0.880	0.120	0.000
Black	31		0.145	0.742	0.225	0.032
White	18		0.528	0.111	0.722	0.167
				**TT**	**GT**	**GG**
Red	50	**c.748T>G**[a), b)]	0.940	0.000	0.120	0.880
Black	31		0.822	0.065	0.225	0.709
White	18		0.444	0.222	0.667	0.111
				**TT**	**TA**	**AA**
Red	50	**c.770T>A**[a)]	0.000	1.000	0.000	0.000
Black	31		0.097	0.806	0.193	0.000
White	18		0.000	1.000	0.000	0.000

[a)] Representing missense mutations, [b)] Representing mutation that being calculate association tests
